# Targeting Obesity and Diabetes to Treat Heart Failure with Preserved Ejection Fraction

**DOI:** 10.3389/fendo.2017.00160

**Published:** 2017-07-17

**Authors:** Raffaele Altara, Mauro Giordano, Einar S. Nordén, Alessandro Cataliotti, Mazen Kurdi, Saeed N. Bajestani, George W. Booz

**Affiliations:** ^1^Institute for Experimental Medical Research, Oslo University Hospital and University of Oslo, Oslo, Norway; ^2^KG Jebsen Center for Cardiac Research, Oslo, Norway; ^3^Department of Pathology, School of Medicine, University of Mississippi Medical Center, Jackson, MS, United States; ^4^Department of Medical, Surgical, Neurological, Metabolic and Geriatrics Sciences, University of Campania “L. Vanvitelli”, Caserta, Italy; ^5^Bjørknes College, Oslo, Norway; ^6^Faculty of Sciences, Department of Chemistry and Biochemistry, Lebanese University, Hadath, Lebanon; ^7^Department of Ophthalmology, School of Medicine, University of Mississippi Medical Center, Jackson, MS, United States; ^8^Department of Pharmacology and Toxicology, School of Medicine, University of Mississippi Medical Center, Jackson, MS, United States

**Keywords:** metabolic disease, heart function, diastolic dysfunction, endothelial and microvascular dysfunction, inflammation, hypertension

## Abstract

Heart failure with preserved ejection fraction (HFpEF) is a major unmet medical need that is characterized by the presence of multiple cardiovascular and non-cardiovascular comorbidities. Foremost among these comorbidities are obesity and diabetes, which are not only risk factors for the development of HFpEF, but worsen symptoms and outcome. Coronary microvascular inflammation with endothelial dysfunction is a common denominator among HFpEF, obesity, and diabetes that likely explains at least in part the etiology of HFpEF and its synergistic relationship with obesity and diabetes. Thus, pharmacological strategies to supplement nitric oxide and subsequent cyclic guanosine monophosphate (cGMP)—protein kinase G (PKG) signaling may have therapeutic promise. Other potential approaches include exercise and lifestyle modifications, as well as targeting endothelial cell mineralocorticoid receptors, non-coding RNAs, sodium glucose transporter 2 inhibitors, and enhancers of natriuretic peptide protective NO-independent cGMP-initiated and alternative signaling, such as LCZ696 and phosphodiesterase-9 inhibitors. Additionally, understanding the role of adipokines in HFpEF may lead to new treatments. Identifying novel drug targets based on the shared underlying microvascular disease process may improve the quality of life and lifespan of those afflicted with both HFpEF and obesity or diabetes, or even prevent its occurrence.

## Introduction

Heart failure (HF) is a major public health problem on a global scale. Historically, HF was believed to originate from long standing systolic dysfunction, as assessed by reduced ejection fraction (HFrEF), and much progress has been made in the last several decades in slowing the inevitably fatal progression of this condition with drugs and in some cases implantable devices (1–5). However, nearly as many individuals are now recognized to exhibit signs of HF, namely dyspnea, fatigue, fluid retention, and exercise intolerance, but yet have a normal or near normal ejection fraction (6–11). This condition of HF with preserved ejection fraction (HFpEF) is thought to be more common in women and more prevalent in the elderly, with similar mortality rates as HFrEF (12–15). HFpEF is documented as the leading cause of hospital admission in patients over 65 years of age and is predicted to be the leading cause of HF within a decade (16, 17). Notably, HFpEF is a leading cause of pulmonary hypertension (HTN) (18).

**Figure 1:**
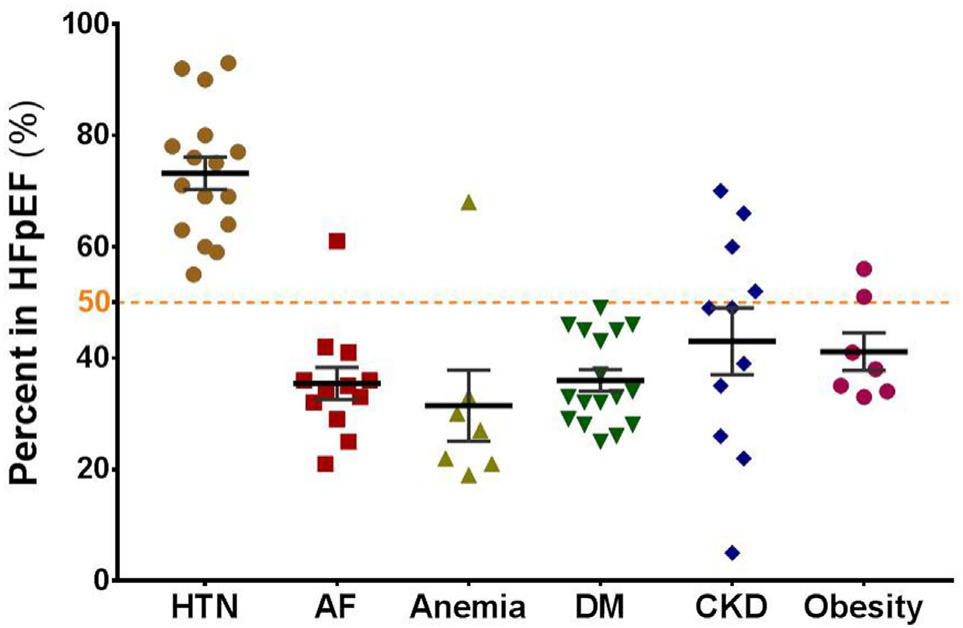
Major comorbidities that negatively affect prognosis in patients with HFpEF. The graph shows the prevalence of comorbidities (in percent) in HFpEF patients enrolled in different clinical studies as summarized by Triposkiadis et al. (35): hypertension (HTN), atrial fibrillation (AF), anemia, diabetes mellitus or type II diabetes (DM), chronic kidney disease (CKD), obesity.

Diastolic dysfunction or impaired relaxation of the left ventricle (LV) is the common clinical condition of HFpEF and is attributable to both cardiac fibrosis and myofilament stiffness (19, 20). Contrary to expectations, recent clinical studies have failed to demonstrate the benefits offered by drugs effective in HFrEF to HFpEF patients (16, 21–23). Thus, HFpEF is one of the largest unmet needs in cardiovascular medicine, and there is a substantial requirement for new therapeutic approaches and strategies that target mechanisms specific for HFpEF (16). A general feature in HFpEF patients is the presence of several comorbidities (Figure 1) including HTN, anemia, atrial fibrillation (AF), obesity, and diabetes (7, 14, 16, 24–30). Moreover, comorbidities negatively affect prognosis to a greater extent in individuals with HFpEF than with HFrEF and have a greater impact on physical impairment as well (31). These observations support the proposition that aggressively targeting comorbidities may prove a more efficacious approach in the clinical management of HFpEF (32–34).

Approximately 50% of patients with HFpEF are obese (35), and HFpEF patients with an increased body mass index (BMI) ≥35 kg/m^2^ are at an increased risk of an adverse outcome (death or cardiovascular hospitalization), independent of other key prognostic variables (36). Obesity is an identified risk factor for HFpEF (28, 37, 38). In a recent study on patients with HFpEF, Dalos et al. (39) found that one-third of patients over a 2-year follow-up reached the combined endpoint of HF hospitalization or cardiac death, which confirms the adverse prognosis of HFpEF. NYHA class III or IV was a strong independent predictor of outcome, along with N-terminal pro-brain natriuretic peptide (NT-proBNP). Correlates of worse NYHA class included NT-proBNP, age, increased values for diastolic dysfunction, and diastolic pulmonary artery pressure. The most novel finding was that BMI was strongly associated with worse NYHA class. The investigators also concluded that a critical contributor to symptoms of breathlessness in patients with HFpEF is increased BMI. Obesity is likely more than a prominent comorbidity for HFpEF and critically involved in its pathogenesis. Increased adiposity promotes HTN, systemic inflammation, insulin resistance, and dyslipidemia, all of which are commonly observed in patients with HFpEF (40). Obesity also impairs cardiac, vascular, and skeletal muscle function (41, 42). Adipose tissue is metabolically active and produces inflammatory cytokines or adipokines, and a number of cardiovascular active substances. Growing evidence reveals that obesity-related microvascular dysfunction, which affects all organs, contributes to exercise-intolerance, and predisposes to the development of microvascular dementia, coronary microvascular angina, chronic obstructive pulmonary disease, pulmonary HTN, and chronic kidney disease (43).

Obesity and diabetes are present in HFpEF patients with a similar proportion (35, 44). In the absence of coronary artery disease and HTN, maladaptive cardiac remodeling associated with diabetes is properly referred to as diabetic cardiomyopathy (35, 45, 46). Accumulating evidence supports the notion that there are two distinct HF phenotypes associated with diabetic cardiomyopathy. Type 1 diabetes leads to HFrEF with a dilated left ventricular phenotype. In contrast, type 2 diabetes, which is a common outcome of obesity, is associated with HFpEF and concentric remodeling of the LV. Seferović and Paulus recently presented evidence attributing the etiology of the two phenotypes to the differential principal involvement of either microvascular endothelial cells (HFpEF) or cardiac myocytes (HFrEF) in the remodeling process (45). An ancillary study of the RELAX (Phosphodiesterase-5 Inhibition to Improve Clinical Status and Exercise Capacity in Diastolic Heart Failure) trial indicated that compared to non-diabetic HFpEF patients, those with diabetes were younger, more obese and more often male, with a higher prevalence of renal dysfunction, HTN, pulmonary disease, and vascular disease (47). Analysis of the I-Preserve [Irbesartan in heart failure with preserved ejection fraction (HFpEF)] trial showed that HFpEF patients with diabetes had more signs of congestion, worse quality of life, and a poorer prognosis with a higher risk of cardiovascular mortality and hospitalization (48). On the basis of 11 clinical features, HFpEF patients who were enrolled in the I-Preserve or CHARM-Preserved (effects of candesartan in patients with chronic HF and preserved left-ventricular ejection fraction) trials were found to fall into one of six subgroups; patients with obesity and or diabetes constituted a distinctive subgroup with (along with another subgroup characterized by advanced age) the worst event-free survival (49).

The goal of our review is to highlight developments in our understanding of obesity- and diabetes-related HFpEF achieved in the last five years. Given the broad magnitude, multifaceted, and syndrome-like nature of the problem, this review is not intended to provide a comprehensive overview of obesity or diabetes and HFpEF. For instance, we do not discuss molecular signaling pathways in cardiac myocytes that are linked to hypertrophy, likely downstream of the initiating stress event (50), or that cause stiffness of myofilaments (51). We do not discuss signaling events in cardiac fibroblasts involved in collagen synthesis or turnover and fibrosis (52); nor do we deal with the importance of skeletal muscle abnormalities in HFpEF (53). Rather, we have chosen to focus on microvascular endothelial dysfunction, based on the compelling evidence that HFpEF is a manifestation of systemic vascular inflammation (54), before discussing potential pharmacological approaches (Table 1).

**Table 1:** Potential targets or approaches for HFpEF.

**Exercise and lifestyle modifications**
Aerobic exercise training
Reduced calorie intake
**Nitric oxide enhancement or replenishment**
Nitroxyl donors
Inorganic nitrates/nitrites
β3 adrenergic receptor agonists
sGC stimulators
**Endothelial cell mineralocorticoid receptor signal**
Spironolactone
**Non-coding RNAs**
AngiomiRs
**Glucose lowering drugs**
Metformin
GLP-1 receptor agonists
SGLT-2 inhibitors
**Novel approaches**
**Enhancing protective guanylyl cyclase systems** LCZ696 PDE9 inhibitors
**Independent of cyclic GMP** ProANP_31–67_

## Role of Coronary Microvascular Inflammation

Microvascular disease appears to be a common feature of obesity, type 2 diabetes, and HFpEF. It is now recognized that obesity is associated with chronic, low-grade systemic vascular inflammation that encompasses the coronary microvasculature and entails impaired angiogenesis, microvascular rarefaction, as well as endothelial dysfunction and impaired vasodilation due to reduced endothelial nitric oxide synthase (eNOS) activity (55–60). Increased circulating levels of adipokines and cytokines contribute to the inflammatory state (57, 59–61). Similarly, both macro- and microvascular derangements are prominent in patients with type 2 diabetes (62, 63), encompassing as well inflammation, endothelial dysfunction, hypercoagubility, functional disruption of the endothelium, rarefaction, and impaired angiogenesis. Also, individuals with type 2 diabetes mellitus suffer from a higher incidence of coronary heart disease as observed in obese patients (64–66).

**Figure 2:**
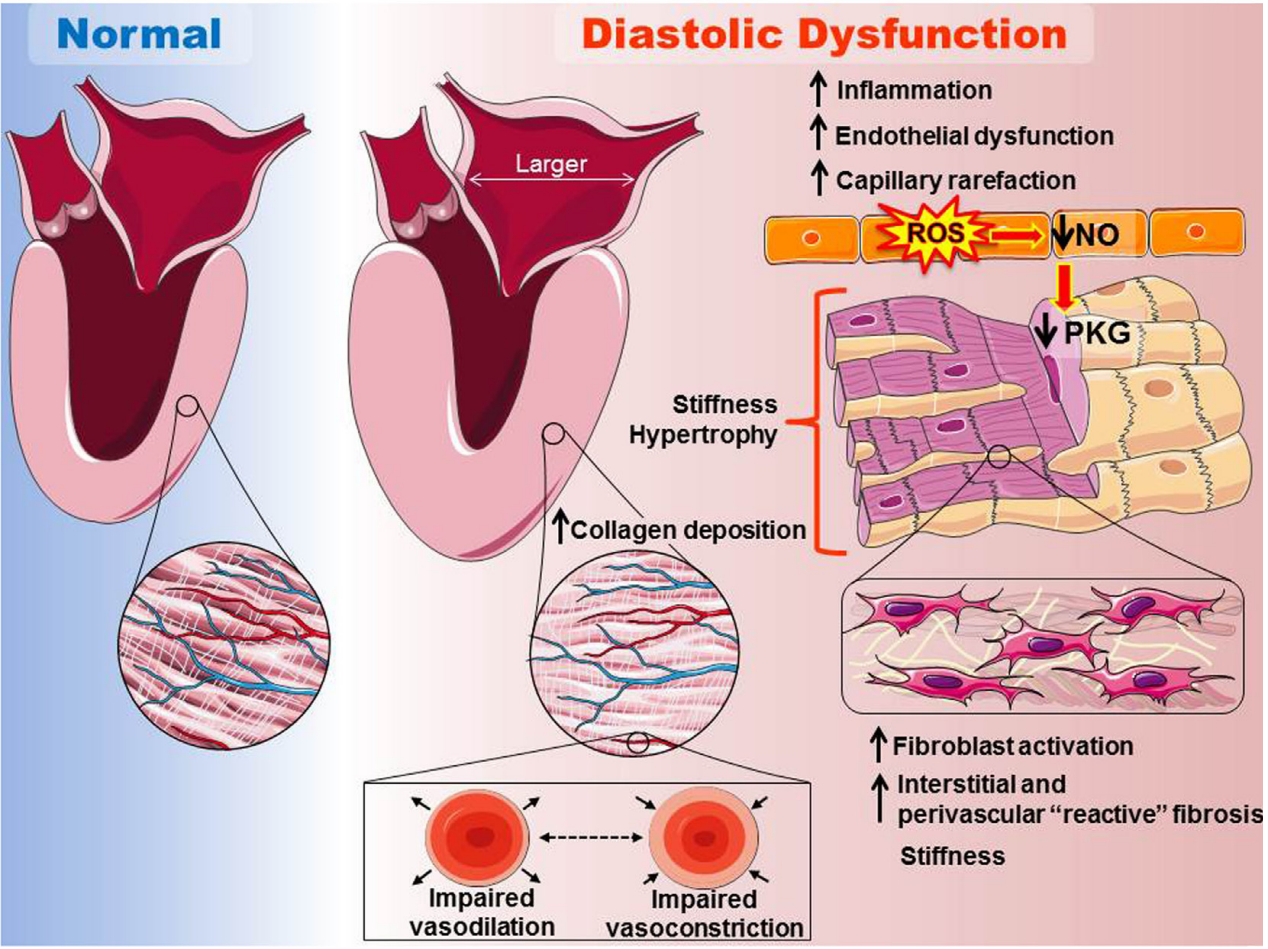
Scheme for the proposed etiology of heart failure with preserved ejection fraction (HFpEF) relevant to obesity and type 2 diabetes. Left side: at the organ level, HFpEF is characterized by cardiac hypertrophy and a marked increase in the left ventricular mass/volume ratio (concentric remodeling), as well as increased stiffness and often enlargement of the left atrium. Right panel: coronary microvascular inflammation is postulated to play a key role in HFpEF progression, encompassing endothelial dysfunction and reduced nitric oxide (NO)-cyclic guanosine monophosphate (cGMP)—protein kinase G (PKG) signaling. Increased stiffness of both myofilaments and the extracellular matrix is thought to impair diastolic function of the heart. The former is postulated to result in part from reduced PKG-mediated phosphorylation of titin, the protein that determines passive elasticity of cardiomyocytes. The later would result from increased collagen deposition and cross-linking (fibrosis), due to loss of cGMP/PKG anti-fibrotic signaling and increased inflammatory endothelium-mediated recruitment of immune cells that activate resident cardiac fibroblasts. Diastolic dysfunction is likely an antecedent event that interacts synergistically with other remodeling events at the cellular level to foster development of HFpEF (images adapted and reproduced with permission from the copyright holder http://servier.com/Powerpoint-image-bank).

Coronary microvascular inflammation is now postulated to play the key role in HFpEF progression, encompassing endothelial dysfunction and impaired nitric oxide (NO)-cyclic guanosine monophosphate (cGMP)—protein kinase G (PKG) signaling and increased collagen deposition (Figure 2) (54). Increased stiffness of both myofilaments and extracellular matrix is thought to impair diastolic function (15, 54, 67). The former is postulated to result from reduced PKG-mediated phosphorylation of titin (20, 54, 67), the protein that determines passive elasticity of cardiomyocytes, and the latter from increased collagen deposition and cross-linking (fibrosis) due to inflammatory endothelium-mediated recruitment of immune cells that activate resident cardiac fibroblasts (15, 20, 67, 68). Diastolic dysfunction is likely an antecedent event that interacts synergistically with other remodeling events at the cellular level to foster development of HFpEF. Recently, levels of inflammatory cells in endomyocardial biopsy samples from HFpEF patients were found to positively correlate with diastolic dysfunction (69) and coronary microvascular dysfunction was detected by angiography in patients with HFpEF (70). Further support for the involvement of myocardial microvascular inflammatory endothelial activation in the etiology of HFpEF comes from a study by Franssen et al. (71). These investigators reported that the myocardium of both HFpEF patients and an obesity-diabetic rat model of HFpEF showed upregulation of endothelial adhesion molecules, elevated expression of the pro-oxidant protein NOX2 in macrophages and endothelial cells but not cardiomyocytes, evidence of the uncoupling of eNOS, and reduced myocardial nitrite/nitrate concentration, cGMP content, and PKG activity.

Involvement of microvascular inflammation in HFpEF with the associated reduction in eNOS-mediated NO generation raises the possibility that enhancing cGMP-PKG signaling could be an efficacious therapeutic approach (46, 72). Potentially, this could be achieved with nitroxyl (HNO), the 1 electron-reduced congener of NO that has myocardial antihypertrophic and superoxide suppressing activity (73, 74), as well as anti-inflammatory actions on microvascular endothelial cell (75). Nitroxyl was also recently shown to inhibit TNF-induced endothelial cell and monocyte activation, as well as leukocyte adhesion to the endothelium, in isolated mouse aorta (76). Nitroxyl increases vasorelaxation and enhances cardiac contractility with positive inotropic and lusitropic effects due to a direct effect on cardiac myofilament proteins and enhancement of SERCA2a activity (77, 78). Nitroxyl may also substitute for NO in activating soluble guanylate cyclase (sGC) and increasing cGMP (79). Recently, chronic treatment with the nitroxyl donor 1-nitrosocyclohexyl acetate was found to attenuate left ventricular diastolic dysfunction in a mouse model of diabetes (80). Others have recently reported evidence indicating that inorganic nitrates and nitrites, which can be converted to NO in the body, are effective in alleviating some HFpEF symptoms (81–84). Lastly, both cardiac myocytes and endothelial cells express the third isotype of beta adrenergic receptors (β3 ARs), which couple to eNOS activation and anti-oxidant signaling (85, 86). Pre-clinical evidence suggests that β3 AR agonists, such as mirabegron, confer protection against diabetes-induced vascular dysfunction and may prove beneficial in HFpEF (85–88).

## Potential Targets or Approaches

### Exercise and Lifestyle Modifications

Preclinical studies have demonstrated beneficial effects of exercise training to protect the heart in obese or diabetic animals. For instance, exercise was reported to protect the hearts of obese diabetic mice from ischemia-reperfusion injury (89) and to reverse cardiac microvascular rarefaction and impaired endothelium-dependent microvascular reactivity in obese diabetic rats (90). In patients with type 2 diabetes, exercise training was reported to improve brachial artery endothelial function (91), as well as to attenuate capillary rarefaction and improve microvascular vasodilator and insulin signaling (92). In contrast, Schreuder et al. (93) did not find any improvement in endothelial function after 8 weeks of training in type 2 diabetes patients. Although a reasonable supposition, there is insufficient data to assess whether dietary and lifestyle changes offer real promise to human sufferers of HFpEF. After all, exercise intolerance is a dominant symptom of HFpEF that contributes in a major way to reduced quality of life in these patients; plus, diabetes has the associated confounding factor of myocardial metabolic inflexibility (45). In a meta-analysis of randomized control trials, physical exercise was found to improve peak oxygen uptake and quality of life in HFpEF patients; however, no significant changes in LV systolic and diastolic function were noted (94). In older adults with type 2 diabetes and chronic renal insufficiency, a moderate protein diet showed long-term effects on low-grade inflammation, and oxidative stress (95), while in elderly HFpEF patients, exercise improved peak exercise oxygen consumption, although endothelial function or arterial stiffness were not altered (96). Among obese older patients with clinically stable HFpEF, caloric restriction or aerobic exercise training increased peak oxygen consumption, and the effects appeared to be additive (97); however, neither intervention had a significant effect on quality of life as assessed by the Minnesota Living with Heart Failure Questionnaire, suggesting that the patients may still have exhibited exertional dyspnea. In any event, no improvements in cardiac function were noted and improvements in peak exercise oxygen consumption were likely due to non-cardiac peripheral adaptations (97, 98). Indeed, sarcopenic obesity may be a significant contributor to exercise intolerance in elderly HFpEF patients (99). Sustained and substantial weight loss *via* bariatric surgery, which was shown effective in improving left ventricular relaxation and reversing concentric LV remodeling and hypertrophy, might be considered to treat obesity-associated HFpEF in younger individuals; however, the long-term cardiovascular effects of this surgery in obese HFpEF patients would need to be assessed (33, 100).

In any case, acute exercise may serve as an important tool for detecting coronary microvascular dysfunction, which becomes more apparent when the heart is challenged in this manner (101). Additionally, exercise would cause the release of a number of hormones or cytokines in HFpEF patients that might impact on cardiac or microvascular function, an area of research that requires further exploration. Recently, exercise training was reported to increase ghrelin levels in patients with HFpEF, especially in patients with higher baseline adiponectin (102). Ghrelin is a gastric hormone that simulates appetite and is associated with weight gain. However, ghrelin was also reported to decrease blood pressure and increase cardiac output in health men (103) and to inhibit apoptosis of cardiomyocytes and endothelial cells *in vitro* (104). Levels of ghrelin are reduced in both obesity and type 2 diabetes (105). Irisin is a novel hormone (myokine) secreted by cardiac and skeletal myocytes in response to exercise that may regulate metabolism and limit weight gain, although its precise role is controversial (106, 107). Circulating levels of irisin are reported to be reduced or increased in obese subjects, but reduced in type 2 diabetic patients (106, 108, 109). Lower levels of irisin are associated with endothelial dysfunction (109, 110). Recently, irisin was found to improve endothelial function in obese mice *via* the activating 5′ adenosine monophosphate-activated protein kinase (AMPK)-eNOS pathway (110); in the spontaneously hypertensive rat, irisin-induced improvement in endothelial function, reduced blood pressure (111).

### Endothelial Cell Mineralocorticoid Receptors Antagonism

Higher circulating aldosterone levels are observed in obesity (112) and type 2 diabetes (113). Moreover, aldosterone antagonism has proven effective in the clinical management of HFrEF (114, 115) and in attenuating cardiac dysfunction and maladaptive remodeling in pre-clinical animal models of obesity-associated HFpEF (116, 117). Surprisingly, the Treatment of Preserved Cardiac Function Heart Failure with an Aldosterone Antagonist (TOPCAT) study, a large randomized, double-blind clinical trial of spironolactone versus placebo in patients with symptomatic HFpEF, did not achieve a significant reduction in the primary composite outcome of time to cardiovascular death from cardiovascular causes, aborted cardiac arrest, or hospitalization for management of HF; however, TOPCAT did demonstrate that spironolactone decreases HF hospitalizations in HFpEF patients (118). Use of spironolactone for HFpEF was associated with an improvement in HF-specific health-related quality of life (119) and, in a separate study, improved exercise tolerance (120). Actually, the beneficial effects of spironolactone in HFpEF may be more significant. Subgroup analysis of TOPCAT by geographic region raised concerns about patient selection and dosing levels in the Russia/Georgia arm of the trial, whereas spironolactone was clearly superior to placebo in reducing cardiovascular events in the Americas (121). Also, spironolactone may have greater potential efficacy in HFpEF patients with lower ejection fraction (122) and, somewhat at odds with this, with lower levels of circulating natriuretic peptides and overall risk (123).

An endothelial-cell targeted strategy may optimize the beneficial actions of aldosterone antagonism in HFpEF. Based on accumulating evidence, Davel et al. recently proposed that in normal physiology, the endothelial mineralocorticoid receptor is vasoprotective; however, in the presence of cardiovascular risk factors, such as obesity and diabetes, endothelial mineralocorticoid receptor activation leads to endothelial dysfunction as a result of reduced eNOS activity and NO production, increased oxidative stress *via* eNOS uncoupling and NOX activation, as well as induced expression of adhesion molecules for inflammatory cells (124). Supporting this possibility is the observation that endothelial mineralocorticoid receptor deletion prevents obesity-induced diastolic dysfunction in female mice (125).

### Non-Coding RNAs

MicroRNAs (miRNAs) are small non-coding RNAs (~21–25 nucleotides in length) that in animal cells generally bind to the 3′ UTR of mRNA to suppress gene expression by either transcript degradation or translational inhibition. The bloodstream contains multiple types of miRNAs in various types of vesicles and complexes, secreted from both healthy and dying cells of likely all tissues throughout the body (126). Since miRNA expression is dynamically regulated, circulating miRNAs are increasingly recognized as having potential utility for diagnostic and prognostic purposes. Recent reports have supported the diagnostic value of using circulating miRNA profiles to distinguish HF patients from non-HF controls and differentiating between HFrEF and HFpEF (127, 128). To date, miRNA profiles have not been defined for HFpEF patients on the basis of dominate comorbidity such as obesity or diabetes. However, both metabolic syndrome and type 2 diabetes are associated with altered circulating miRNA profiles (126, 129). The endothelium is a rich source of circulating miRNAs in both the healthy and disease states and the plasma mRNA profile provides an assessment of endothelial health (126). For instance, circulating and cardiac levels of pro-angiogenic miR-126 and miR-132 were found to be downregulated in type 2 diabetic individuals without any known history of cardiovascular disease (130). Decreased levels of these miRNAs were associated with cardiac microangiopathy as indicated by reduced capillaries and arterioles and increased endothelial cell apoptosis. Parallel findings in a mouse model of type 2 diabetes support the prognostic value of these “angiomiRs”. Interestingly, swimming training in rats was reported to increase cardiac miRNA-126 expression and angiogenesis (131). Optimistically, the identification of particular miRNA signature in diabetes- or obesity-associated HFpEF could lead to miRNA-based therapies that use tissue-targeted exosomes to deliver anti-miRNA or miRNA mimics to treat microvascular dysfunction. Some of the challenges in making miRNA-based therapy a reality are discussed elsewhere (132–134).

An emerging area in cardiovascular medicine is the study of long non-coding RNAs (lncRNAs), which are transcripts larger than 200 nucleotides that control gene expression at the epigenetic, transcriptional, and posttranscriptional levels (135). Single-nucleotide polymorphisms which alter the expression of the lncRNA ANRIL (antisense non-coding RNA in the INK4 locus) are associated with coronary artery disease and type 2 diabetes (126). Recently, circulating levels of three lncRNAs were identified as biomarkers of diastolic function and remodeling in patients with well-controlled type 2 diabetes (136): long intergenic non-coding RNA predicting cardiac remodeling (LIPCAR), myocardial infarction-associated transcript (MIAT), and endothelial cell-enriched migration/differentiation-associated long non-coding RNA (SENCR). Although the cellular source was not defined in this study, LIPCAR is thought to originate from cardiomyocyte mitochondria, whereas MIAT and SENCR have been implicated in endothelial cell function/dysfunction, including inflammation and angiogenesis (126, 136, 137). The role and diagnostic/prognostic value of lncRNAs in obesity or diabetes associated HFpEF awaits investigation.

### Glucose Lowering Drugs

The drug metformin has proven highly effective in the treatment of type 2 diabetes and is currently recommended as first line treatment. Metformin has beneficial actions by reducing hepatic glucose production and by activating AMPK, which enhances cellular glucose uptake. AMPK activation in cardiac myocytes may also inhibit hypertrophy (138). Preclinical studies demonstrated that AMPK activation by metformin restores endothelial function and NO bioavailability by attenuating oxidative and endoplasmic reticulum stress and by directly increasing eNOS activity (139, 140). However, metformin does not seem to improve LV stiffness in type 2 diabetic patients (141).

Concerns of increased adverse cardiovascular outcomes, including HF, are associated with the use of sulfonylureas and thiazolidinediones (TZDs) in diabetic patients (45, 142, 143). The situation with regard dipeptidyl peptidase-4 inhibitors is unsettled (144). Although glucagon-like peptide-1 (GLP-1) receptor agonists, liraglutide and semaglutide, showed a reduction in cardiovascular events, GLP-1 agonists do not seem to have a significant effect on natriuretic peptide levels in HF (45, 145). Much excitement has been generated by the recent approval of selective sodium glucose transporter 2 (SGLT-2) inhibitors, including empagliflozin, to treat type 2 diabetes. SGLT-2 inhibitors lower blood glucose by blocking sodium-dependent reabsorption of glucose in the proximal tubule and causing glycosuria. However, the beneficial actions of SGLT-2 inhibitors in type 2 diabetes seem to extend beyond glycemic control and are not completely understood (146). SGLT-2 inhibitors are associated with weight loss and reductions in blood pressure (without an increase in heart rate), visceral adiposity, plasma urate levels, and arterial stiffness/vascular resistance, as well as improvements in microvascular/macrovascular endothelial function and cardiac metabolism (146). The recently published results of the EMPA-REG OUTCOME trial revealed a marked reduction in deaths from cardiovascular causes, HF hospitalizations, and deaths from any cause when empagliflozin was added to standard care of patients with type 2 diabetes (147). At present, insufficient evidence precludes reaching a definitive conclusion as to whether the beneficial effects of empagliflozin represent a class effect of SGLT-2 inhibitors (148).

### Novel Approaches That Enhance Guanylyl Cyclase Systems

Nitric oxide deficiency is postulated to be responsible for diastolic dysfunction in HFpEF patients due to impaired cGMP generation and PKG activation. Because of issues such as tolerance and preload reduction, organic nitrates seem not to be useful in treating HFpEF (149). Alternative ways of cGMP enhancement might hold more promise for future therapeutic benefit. sGC stimulators are a relatively new class of drugs that act *via* an allosteric site on sGC to synergize with NO in producing cGMP, thereby offsetting decreased NO due to diminished NO synthase activity (150). The recently completed phase II SOluble guanylate Cyclase stimulatoR in heArT failurE Study (SOCRATES) program consisted of two parallel studies to assess the potential utility of the sGC stimulator, vericiguat for treating HFrEF (SOCRATES-REDUCED) and HFpEF (SOCRATES-PRESERVED). The respective primary endpoints were change in NT-proBNP at 12 weeks, and change in NT-proBNP and left atrial volume at 12 weeks (151). Vericiguat was well tolerated; however, likely because of inadequate dosage level, SOCRATES-REDUCED yielded mixed, yet promising results (152). The outcome of SOCRATES-PRESERVED has not been reported but likely is complicated by the same shortfall in dosing as the SOCRATES-REDUCED study.

Alternative NO-independent ways to increase cGMP formation, which is linked to anti-hypertrophy and anti-fibrosis signaling in the heart (153, 154), may prove beneficial in treating HFpEF. Specifically, receptors for natriuretic peptides activate membrane-bound particulate GC. Indeed, several studies have shown favorable cardiorenal effects, including improvement of diastolic function, of exogenous supplementation of the natriuretic peptides, which are known to stimulate cGMP production in the heart, kidney, and vasculature (155, 156). In contrast, deletion of the BNP gene is characterized by diastolic dysfunction, cardiac remodeling, and rising of elevated blood pressure (157). A recently approved drug for the treatment of chronic HF, LCZ696 (brand name entresto), combines an angiotensin II type 1 receptor blocker (valsartan) with a neprilysin inhibitor (sacubitril). Sacubitril suppresses proteolysis of natriuretic peptides that enhance cGMP signaling independent of NO (158). The phase III study Efficacy and Safety of LCZ696 Compared to Valsartan, on Morbidity and Mortality in Heart Failure Patients With Preserved Ejection Fraction (PARAGON-HF) (NCT01920711) is currently underway, while preliminary data from the PARAMOUNT study have shown a significant reduction of the circulating levels of NT-proBNP (a major prognostic biomarker in HF) after 12 weeks of treatment, and an improvement of both cardiac size and New York Heart Association (NYHA) class at 36 weeks as compared to valsartan (159). Selective inhibitors of phosphodiesterase-9 (PDE9), which hydrolyzes natriuretic peptide-coupled cGMP and is upregulated in HFpEF, are another potential way to increase cardiac cGMP levels (160).

Inadequate processing and activation of natriuretic peptides appears to be a signature of HTN, resulting in an impaired counter-regulatory response of the natriuretic homeostatic control system (161, 162). Notably, although natriuretic peptides are useful to stratify HFpEF patients in conjunction with the NYHA classification system, circulating levels of BNP are not elevated as much in HFpEF patients as in HFrEF (163, 164). It is now established that elevated circulating natriuretic peptides in patients with overt cardiovascular diseases, although having a significant adverse prognostic value, are constituted mainly of biologically non-active forms, while mature active forms are virtually absent in severe congestive HF patients (165). In addition, obesity has a negative impact on the elevation of circulating levels of BNP as fatty tissue expresses the clearance receptor for the natriuretic peptide (NPRC) (163, 166). Therefore, supplementation of these cardioprotective natriuretic peptides may prove to be of therapeutic importance in obesity- or diabetes-associated HFpEF. Studies report that circulating atrial natriuretic peptide (ANP) can break down into multiple peptides, each of which has distinctive actions. One of these peptides, namely proANP_31–67_, does not activate the cGMP pathway, but exerts a unique cardiac and renal protective response by increasing renal, as well as circulating levels of prostaglandin E2 (PGE2) (167–169). ProANP_31–67_ also has vasodilatory actions and induces diuresis *via* inhibition of the basolateral Na^+^–K^+^ ATPase of the inner medullary collecting ducts resulting in increased Na^+^ and renal water excretion (170, 171). Whether the potential benefits of proANP_31–67_ extend to HFpEF is not established, although PGE2 has protective effects on the heart *via* enhancement of VEGF and eNOS expression levels and anti-inflammatory actions (172, 173). Future studies are warranted to determine whether the cardiorenal protective effects and the cardiac function enhancing properties of these hormones can be explained by mechanisms different from cGMP activation.

## Unresolved Issues

Adipose tissue is an endocrine organ that secretes multiple “adipokines” that have broad physiological and pathological impact throughout the body (174, 175). In obesity, the altered circulating adipokine profile contributes to systemic low-grade inflammation and the cardiovascular or obesity-related comorbidities defining the metabolic syndrome. Understanding the contribution of a particular adipokine to the disease process is a challenging task as the inflammatory milieu is a dynamic and fluid environment of multiple players with redundant or conflicting roles. A good case in point is the role of adiponectin in HFpEF. Adiponectin is the major adipokine produced by adipose tissue with anti-inflammatory, antidiabetic, anti-apoptotic, and anti-atherogenic properties (174, 176). Circulating adiponectin levels are decreased in obesity and type 2 diabetes and downregulation of adiponectin and its receptors is associated with insulin resistance and diabetes, as well as increased risk of HTN and coronary artery disease (174, 176). Animal studies have shown that adiponectin can inhibit cardiac hypertrophy and fibrosis, and reduce infarct size (174). Together these findings support the supposition that adiponectin might have therapeutic potential in HFpEF patients (176). However, circulating adiponectin levels are increased in both HFrEF and HFpEF (177). Furthermore, multiple studies have shown an association between higher adiponectin levels and increased mortality and cardiovascular disease mortality/morbidity in diverse populations (178). One confounding factor is that natriuretic peptides, which are elevated in HF due to hemodynamic stress and/or neurohormonal activation, may directly enhance adiponectin expression (178). Certainly, the question of which-time-point in the development and progression of HFpEF is an important consideration. Sex differences may play a role as well. Low adiponectin was associated with higher odds of indices of diastolic dysfunction in women, but lower odds in men, and lower adiponectin was associated with increased left ventricular mass only in women (179). Other variables that may come into play are adiponectin receptor desensitization, receptor subtypes, and the different-size molecular weight complexes of circulating adiponectin (“isoforms”) (176).

## Perspectives and Future Directions

The role of sex as well as race in HFpEF, especially their interaction with comorbidities, is an evolving area of investigation. Early studies reported that HFpEF was more common among women than men (180, 181). Recently, the largest sex- and race-based subgroup analysis of HFpEF was published, involving data gathered from 1,889,608 hospitalizations (182). The study reported several noteworthy findings, including the following: (a) men with HFpEF were slightly younger than women with HFpEF and had a higher burden of comorbidities; (b) blacks with HFpEF were younger than whites with HFpEF, with lower rates of most comorbidities; (c) HTN, anemia, chronic renal failure, and diabetes, were more common among blacks; (d) AF was an important correlate of mortality only among women and blacks; and (e) with women, chronic pulmonary disease, and diabetes were more common among younger patients, but more common among older patients in men. Obviously, the influence of sex and race in the context of comorbidities to the heterogeneity of HFpEF is complicated and further study is needed. Another emerging area of interest is the additional classification according to the 2016 EC guidelines of HFmrEF, for HF patients exhibiting mid-range ejection fractions (183). The clinical profile, including comorbidities, and prognosis of patients diagnosed with HFmrEF, and the etiological and prognostic relationship of this HF phenotype to HFrEF and HFpEF needs to be addressed. The application of novel measures for assessing LV function such as strain imaging may be useful in this regard.

Obesity and diabetes are not only risk factors for the development of HFpEF but have significant impact on its symptoms and outcome. Therefore, focusing on these comorbid conditions in HFpEF might provide a novel therapeutic strategy. Coronary microvascular endothelial dysfunction with impaired NO-cGMP-PKG signaling is a shared condition that is thought to be the basis for diastolic stiffness, inflammation, oxidative stress, and maladaptive cardiac remodeling. Pharmacological approaches that target this signaling axis offer promise in treating or preventing HFpEF. This would include: (a) NO replenishment (inorganic nitrates/nitrites), replacement (nitroxyl donors), or enhanced generation (β3 AR agonists and AMPK agonist); and (b) enhancers of NO-independent cGMP generation (LCZ696/entresto) or prevention of its breakdown (PDE9 inhibitors). A reappraisal of clinical results supports the utility of inhibiting the mineralocorticoid receptor in treating HFpEF, but additional study is warranted. In addition, given the pronounced side effects of spironolactone at higher doses, an endothelial cell-targeted approach might be judicious. miRNA and lncRNA profiling of HFpEF patients offers the promise of not only prognostic assessment and therapeutic monitoring, but personalized treatment strategies as well. A better understanding of the role of adipokines in obesity- and diabetes-associated HFpEF may open up new pharmacological avenues. Finally, SGLT-2 inhibitors offer great promise for treating or preventing HFpEF in obese and diabetic patients. A better understanding of the physiological and molecular basis for the cardiovascular protective actions of this new drug class should foster the development of even more effective compounds.

## Author Contributions

All authors contributed conceptually to the manuscript. All authors authored sections of the manuscript, contributed to figure design, and approved the final version. All appropriate permissions have been obtained from the copyright holders of any work that has been reproduced in this manuscript.

## Conflict of Interest Statement

The authors declare that the research was conducted in the absence of any commercial or financial relationships that could be construed as a potential conflict of interest.
